# Impact of Punctate Hyperfluorescence Status on Treatment Outcomes of Faricimab Versus Aflibercept in Neovascular Age-Related Macular Degeneration

**DOI:** 10.3390/jcm14186637

**Published:** 2025-09-20

**Authors:** Hiroyuki Kamao, Katsutoshi Goto, Kenichi Mizukawa, Ryutaro Hiraki, Atsushi Miki, Shuhei Kimura

**Affiliations:** 1 Department of Ophthalmology, Kawasaki Medical School, 577 Matsushima, Kurashiki 701-0192, Japan; k_goto@med.kawasaki-m.ac.jp (K.G.); hiraki@med.kawasaki-m.ac.jp (R.H.); amiki@med.kawasaki-m.ac.jp (A.M.); kimuras@med.kawasaki-m.ac.jp (S.K.); 2 Shirai Eye Hospital, 1339 Takasecho Kamitakase, Mitoyo 767-0001, Japan; mizu-p@shirai-hosp.or.jp

**Keywords:** neovascular age-related macular degeneration, faricimab, aflibercept, punctate hyperfluorescence, angiopoietin-2

## Abstract

**Background/Objectives**: To compare the treatment outcomes of intravitreal faricimab (IVF) and intravitreal aflibercept (IVA) in treatment-naïve patients with neovascular age-related macular degeneration (nAMD), stratified by the presence or absence of punctate hyperfluorescence (PH). **Methods**: This retrospective study included 301 treatment-naïve patients with nAMD who underwent either IVF or IVA. After 1:1 propensity score matching based on baseline best-corrected visual acuity (BCVA), age, and PH status, 56 eyes (28 per group) were analyzed within each PH subgroup. Outcome measures included BCVA, central retinal thickness (CRT), subfoveal choroidal thickness (SFCT), and no retinal fluid rate during the loading dose regimen, and the retreatment rate after the loading dose regimen. The prespecified primary endpoint was the 1-year retreatment rate after completion of the loading dose regimen, analyzed by Kaplan–Meier curves with log-rank tests. Comparisons were performed separately between the PH and non-PH groups. **Results**: In the PH group, no significant differences were observed between IVF and IVA groups in terms of BCVA, CRT, SFCT, no retinal fluid rate, or retreatment rate at any time point. In the non-PH group, IVF and IVA groups showed no significant differences in BCVA, CRT, or SFCT at any time point; however, the IVF group achieved a significantly higher no retinal fluid rate (100.0% vs. 64.3%, *p* < 0.001) and a lower retreatment rate at 1 year (71.4% vs. 92.9%, *p* = 0.004) than the IVA group. **Conclusions**: IVF and IVA showed comparable efficacy in nAMD with PH. In contrast, IVF demonstrated superior anatomical outcomes in nAMD without PH. These retrospective findings suggest distinct pathophysiological mechanisms between PH and non-PH subtypes.

## 1. Introduction

Neovascular age-related macular degeneration (nAMD) is the leading cause of visual impairment in developed countries [1]. The advent of anti-vascular endothelial growth factor (VEGF) therapy has markedly improved visual outcomes. However, current treatment regimens require frequent intravitreal injections and continuous follow-up visits, imposing substantial time and economic burdens on patients and healthcare providers. Therefore, identifying treatment strategies that achieve no retinal fluid while allowing extended dosing intervals is of paramount clinical importance.

In Japan, anti-VEGF agents approved for the treatment of nAMD include ranibizumab [2], aflibercept [3], brolucizumab [4], and faricimab [5]. Pharmacologically, ranibizumab, aflibercept, and brolucizumab inhibit targets within the VEGF family, whereas faricimab targets both the VEGF family and angiopoietin-2 (Ang-2). Because Ang-2 antagonizes Ang-1/Tie2 signaling and promotes endothelial destabilization [6], dual VEGF-A and Ang-2 blockade is biologically expected to promote no retinal fluid and treatment durability. Consistently, phase III clinical trials have demonstrated that faricimab achieves visual outcomes noninferior to those of aflibercept, with superior anatomical improvements and the added advantage of extended dosing intervals [7]. These findings suggest that faricimab may be particularly beneficial for nAMD subtypes in which Ang-2 contributes to disease pathogenesis. However, the specific patient subgroups most likely to benefit from intravitreal faricimab (IVF) remain to be identified.

Pachychoroid neovasculopathy (PNV) [8], first described by Pang et al., is a form of macular neovascularization (MNV) associated with a pathologically thickened choroid (pachychoroid). Several studies have reported that PNV requires fewer anti-VEGF treatments than non-PNV [9,10]. Punctate hyperfluorescence (PH) is frequently observed in the central region of the choroidal vascular hyperpermeability (CVH) during the mid- to late-phase indocyanine green angiography (ICGA) in most eyes with central serous chorioretinopathy (CSC) [11] and polypoidal choroidal vasculopathy (PCV) [12], both of which are classified as pachychoroid spectrum diseases. The relationship between PH and treatment outcomes following anti-VEGF therapy in patients with nAMD has been previously investigated. It was found that patients with PH, indicative of PNV, had a lower recurrence rate after the initial loading dose of intravitreal aflibercept (IVA) [13] and achieved a higher long-term no retinal fluid rate following a switch to intravitreal brolucizumab in eyes with aflibercept-refractory nAMD [14]. These findings, which are consistent with previous reports, suggest that PNV responds favorably to anti-VEGF therapy. Therefore, the establishment of effective treatment strategies for patients without PH, representing non-PNV, is clinically important.

In the present study, propensity score matching was used to compare the therapeutic outcomes of IVF and IVA in treatment-naïve patients with nAMD, stratified by the presence or absence of PH. This approach aimed to elucidate the clinical efficacy of Ang-2 inhibition, thereby contributing to a better understanding of nAMD pathophysiology and supporting the development of individualized treatment strategies.

## 2. Materials and Methods

### 2.1. Study Design

This study retrospectively reviewed the medical records of all patients with treatment-naïve nAMD who were treated with IVF or IVA at the Kawasaki Medical School between May 2022 and May 2024. Data on hypertension, diabetes, and smoking status were collected from hospital records. Smoking status was categorized as never-smoker or ever-smoker, following the classification used in a previous study [15]. We excluded patients who had undergone laser photocoagulation or vitrectomy, as well as those with macular neovascularization secondary to high myopia (>−6 diopters), uveitis, or angioid streaks. Patients with other ocular diseases that could potentially affect the treatment outcomes in the study eye, such as branch retinal vein occlusion, diabetic retinopathy, or glaucoma, were also excluded.

### 2.2. Treatment Method and Data Collection

All enrolled patients received three consecutive monthly injections of faricimab or aflibercept as a loading dose regimen, followed by monthly monitoring under a pro re nata (PRN) regimen. After completion of the loading dose regimen, eyes with residual or recurrent retinal fluid—defined as subretinal fluid (SRF) and/or intraretinal fluid (IRF) detected on optical coherence tomography (OCT)—were classified into the retreatment group.

All participants underwent a comprehensive ophthalmologic examination, including measurement of best-corrected visual acuity (BCVA), indirect ophthalmoscopy, slit-lamp biomicroscopy with a noncontact lens, color fundus photography, fundus autofluorescence (FAF) (TRC-50DX; Topcon Corporation, Tokyo, Japan), swept-source OCT (DRI OCT-1 Atlantis; Topcon Corporation, Tokyo, Japan), fluorescein angiography (FA), and ICGA (HRA-2; Heidelberg Engineering GmbH, Dossenheim, Germany). Visual acuity was measured in decimal notation and converted to logarithm of the minimum angle of resolution (logMAR) units for analysis. Central retinal thickness (CRT) and subfoveal choroidal thickness (SFCT) were measured using swept-source OCT, as previously described [15]. PH was assessed in all fellow eyes using mid- to late-phase ICGA, based on previous studies [13,14,16]. PH is typically distributed along the choroidal vessels and appears as solitary or clustered hyperfluorescent spots within the CVH areas. Two masked retinal specialists (H.K. and K.G.) independently assessed the presence or absence of PH. Discrepancies were adjudicated by a senior grader (K.M.). Inter-grader agreement in this study was high (Cohen’s κ = 0.96, *p* < 0.0001). Subretinal hyperreflective material (SHRM) in all affected eyes, defined as a hyperreflective signal above the retinal pigment epithelium (RPE) on OCT images, was classified as exudation, hemorrhage, neovascular tissue, vitelliform, fibrosis, or no SHRM based on fundus color photography, FAF, OCT, OCT angiography, FA, and ICGA, as previously reported [13]. None of the eyes in the present study had vitelliform tissue or fibrosis. At the time of PH and SHRM classification, all patients had unilateral MNV. In cases where MNV subsequently developed in the fellow eye and treatment was initiated, the classifications of PH and drusen were determined based on findings from the initially affected eye.

### 2.3. Outcome Measures

The following baseline characteristics were compared between the IVF and IVA groups: age, sex, prevalence of hypertension or diabetes, smoking history, SHRM subtype, presence of IRF and SRF, and presence of polypoidal lesions. Outcome measures included BCVA, CRT, SFCT, the presence or absence of retinal fluid (SRF or IRF) during the loading dose regimen, and the presence or absence of fluid up to 1 year after completion of the loading dose regimen.

### 2.4. Statistical Analysis

Statistical analyses, including propensity score matching, were performed using the JMP Pro 17 software (SAS Institute, Cary, NC, USA). One-to-one propensity score matching was conducted based on age, baseline visual acuity, and the presence or absence of PH. Covariate balance between IVF and IVA was assessed using absolute standardized mean differences (ASMDs). ASMD < 0.10 is considered well balanced. Changes in BCVA, CRT, and SFCT over time (baseline, 1, 2, and 4 months) were analyzed using linear mixed-effects models. For each outcome, fixed effects were drug (IVF vs. IVA), month, and the drug × month interaction; a subject-specific random intercept accounted for repeated measures. Alternative within-subject covariance structures (unstructured [UN] and first-order autoregressive (1) [AR]) were compared using the corrected Akaike information criterion (AICc), and the structure with the lowest AICc was adopted. Models were estimated by restricted maximum likelihood (REML), with Type III tests of fixed effects and Satterthwaite approximation for denominator degrees of freedom. We report least-squares means (LS means) with 95% confidence intervals (CIs). Multiplicity-adjusted pairwise contrasts (Tukey–Kramer) were performed for within-drug month comparisons and between-drug comparisons at each month. Pearson’s chi-square test or Fisher’s exact test, as appropriate, was used to assess the differences in sex distribution, prevalence of hypertension or diabetes, smoking history, distribution of SHRM subtypes, and presences of IRF, SRF, and polypoidal lesions between the IVF and IVA groups. The no retinal fluid rate during the loading dose regimen and the retreatment rate over 1 year were analyzed using Kaplan–Meier methods with log-rank tests; Cox proportional hazards models were used to estimate hazard ratios with 95% CIs. Statistical significance was set at *p* < 0.05. Single and double asterisks (* and **) indicate *p* < 0.05 and *p* < 0.01, respectively.

## 3. Results

### 3.1. Clinical Characteristics in the Study Population

**Figure 1:**
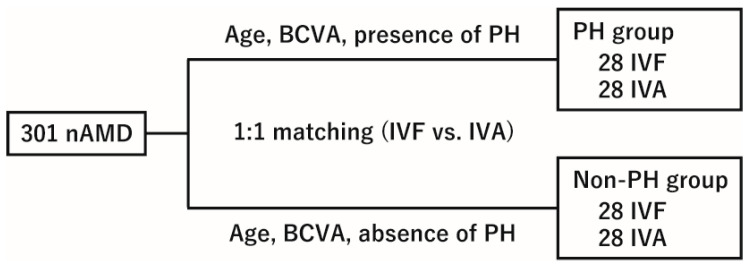
Propensity score matching of patients by age, baseline visual acuity, and PH status. Patient selection and 1:1 propensity score matching between the IVF and IVA groups were adjusted for age, baseline visual acuity, and the presence or absence of PH. nAMD, neovascular age-related macular degeneration; BCVA, best-corrected visual acuity; PH, punctate hyperfluorescence; IVF, intravitreal faricimab; IVA, intravitreal aflibercept.

Of the 301 eligible patients who underwent IVF or IVA, 1:1 propensity score matching was performed based on age, baseline visual acuity, and the presence or absence of PH (PH group or non-PH group) (Figure 1). After matching, the PH and non-PH groups comprised 28 eyes treated with IVF and 28 eyes treated with IVA, with 56 eyes per group. In the PH group, the ASMD decreased from 0.19 to 0.05 for age and from 0.47 to 0.08 for baseline BCVA. In the non-PH group, the ASMD decreased from 0.04 to 0.03 for age, and from 0.57 to 0.01 for baseline BCVA. All post-match ASMDs were <0.10, meeting our prespecified balance criterion. The baseline characteristics are summarized in Table 1. Within both the PH and non-PH groups, no significant differences were found between the IVF and IVA groups in terms of age, sex distribution, prevalence of hypertension or diabetes, smoking history, proportion of SHRM subtypes, or frequencies of IRF, SRF, and polypoidal lesions.

### 3.2. Treatment Outcomes in the Loading Dose Regimen

**Figure 2:**
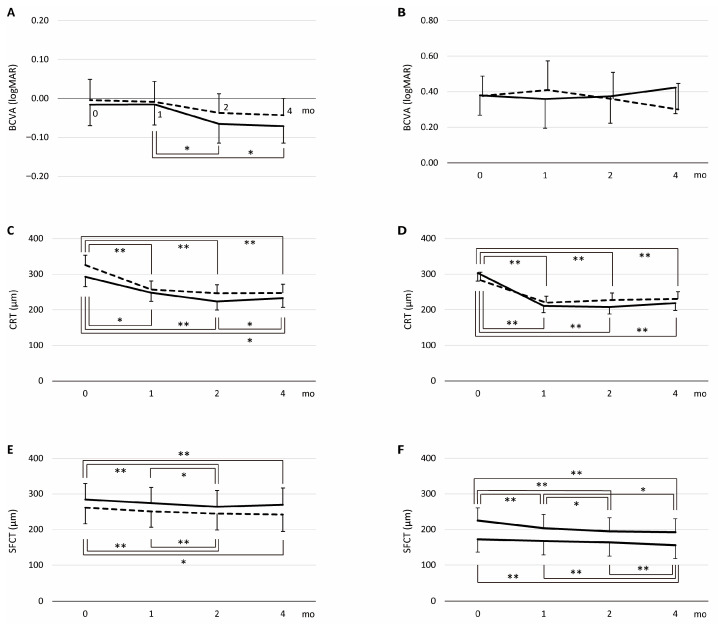
The time course of BCVA, CRT, and SFCT in the loading dose regimen. Time course of adjusted means (LS means ± 95% CI) for logMAR (**A**,**B**), CRT (**C**,**D**), and SFCT (**E**,**F**) at 0, 1, 2, and 4 months during the loading phase. Panels (**A**,**C**,**E**) show the PH group, and panels (**B**,**D**,**F**) show the non-PH group. Solid lines represent the IVF group, and dashed lines represent the IVA group. Brackets with asterisks indicate multiplicity-adjusted pairwise comparisons of LS mean: horizontal brackets, within drug across months; vertical brackets, between drugs within a month. Single and double asterisks (* and **) indicate *p* < 0.05 and *p* < 0.01, respectively. BCVA, best-corrected visual acuity; CRT, central retinal thickness; SFCT, subfoveal choroidal thickness.

**Figure 3:**
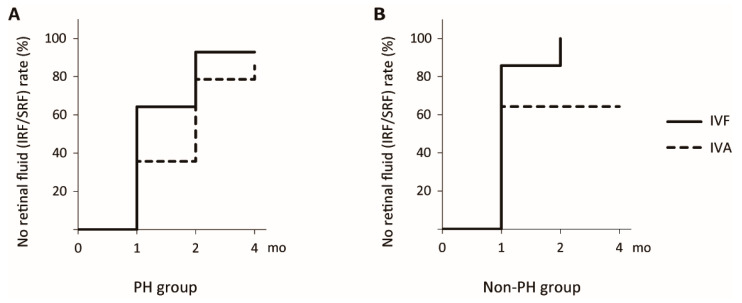
No retinal fluid rate in the loading dose regimen. (**A**) Kaplan–Meier curve for the time to no retinal fluid after the first injection in the PH group. (**B**) Kaplan–Meier curve of the time to no retinal fluid after the first injection in the non-PH group. Solid lines represent the IVF group, and dashed lines represent the IVA group. PH, punctate hyperfluorescence; IVF, intravitreal faricimab; IVA, intravitreal aflibercept.

In the PH group, among alternative covariance structures, the UN model provided the best fit (lowest AICc) for all outcomes: BCVA (−439.84 for UN vs. −436.04 for AR), CRT (2342.27 vs. 2468.43), and SFCT (2108.06 vs. 2173.18); therefore, the UN model was used throughout. Corresponding LS means are summarized in Table 2. For BCVA, Type III tests showed no effect of drug (*p* = 0.55) and no drug × month interaction (*p* = 0.75), with a significant month effect (*p* = 0.003) (Figure 2A). Tukey–Kramer comparisons found no within-drug month differences for the IVA group, whereas the IVF group improved from month 1 to month 2 (*p* = 0.01) and month 4 (*p* = 0.02). For CRT, Type III tests showed a significant month effect (*p* < 0.001), with no effect of drug (*p* = 0.13) and no drug × month interaction (*p* = 0.09) (Figure 2C). Both groups showed early reductions from baseline. In the IVF group, CRT decreased from baseline to months 1, 2, and 4 (*p* < 0.05), and month 2 was lower than month 4 (*p* = 0.02). In the IVA group, CRT decreased from baseline to months 1, 2, and 4 (all *p* < 0.01). For SFCT, Type III tests showed a significant month effect (*p* < 0.001), with no effect of drug (*p* = 0.47) and no drug × month interaction (*p* = 0.06) (Figure 2E). Both groups showed progressive reductions over time. In the IVF group, SFCT decreased from baseline to months 2 and 4 (both *p* < 0.05), and month 2 was lower than month 1 (*p* < 0.01). In the IVA group, SFCT decreased from baseline to months 2 and 4 (both p < 0.01), and month 2 was lower than month 1 (*p* = 0.03). Four months after initial treatment, the no retinal fluid rate was 92.9% (26/28 eyes) in the IVF group and 85.7% (24/28 eyes) in the IVA group. The no retinal fluid rate showed no significant difference between the two groups (log-rank *p* = 0.06; HR = 1.43, 95% CI: 0.82–2.51, *p* = 0.21; Figure 3A).

In the non-PH group, among alternative covariance structures, the UN model provided the best fit for all outcomes: BCVA (−131.62 for UN vs. −31.88 for AR), CRT (2221.09 vs. 2398.40), and SFCT (2143.24 vs. 2196.09); therefore, the UN model was used throughout. Corresponding LS means are summarized in Table 2. For BCVA, Type III tests showed no effect of drug (*p* = 0.81) and no effect of month (*p* = 0.51), with a significant drug × month interaction (*p* < 0.001) (Figure 2B). Tukey–Kramer comparisons detected no between-drug differences at any month; within groups, the IVF group showed no significant month-to-month changes, whereas in the IVA group BCVA at month 4 was lower than month 1 (*p* = 0.01). For CRT, Type III tests showed a significant month effect (*p* < 0.001) and drug × month interaction (*p* = 0.04), with no effect of drug (*p* = 0.69) (Figure 2D). Both groups showed early reductions from baseline. The reduction from baseline to month 1 was numerically larger in the IVF group than in the IVA group (−92 µm vs. −63 µm). For SFCT, Type III tests showed a significant month effect (*p* < 0.001) and drug × month interaction (*p* = 0.03), with no effect of drug (*p* = 0.15) (Figure 2F). Both groups showed progressive reductions over time. The IVA group showed an early reduction from baseline to month 1 (−21.4 µm, *p* = 0.005), whereas the IVF group showed a later reduction with minimal change from baseline to month 1 (−4.9 µm, *p* = 0.85) and a smaller overall reduction by month 4 (−16.6 µm) than IVA (−32.6 µm). Four months after initial treatment, the no retinal fluid rate was 100.0% (28/28 eyes) in the IVF group and 64.3% (18/28 eyes) in the IVA group. The IVF group had a higher no retinal fluid rate than the IVA group (log-rank *p* = 0.001; HR = 1.86, 95% CI: 1.01–3.44, *p* = 0.047; Figure 3B).

### 3.3. Retreatment Rate After the Loading Dose Regimen over 1 Year

**Figure 4:**
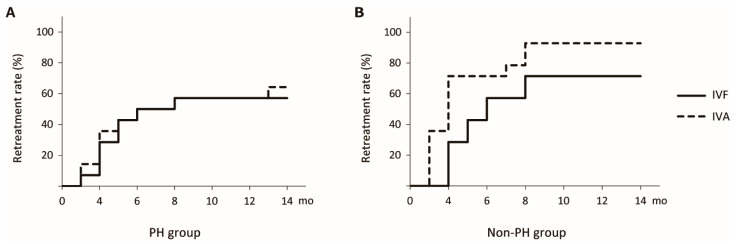
Retreatment rate after the loading dose regimen. (**A**) Kaplan–Meier curve for the time to retreatment after the loading dose regimen in the PH group. (**B**) Kaplan–Meier curve for the time to retreatment after the loading dose regimen in the non-PH group. Solid lines represent the IVF group, and dashed lines represent the IVA group. PH, punctate hyperfluorescence; IVF, intravitreal faricimab; IVA, intravitreal aflibercept.

**Figure 5:**
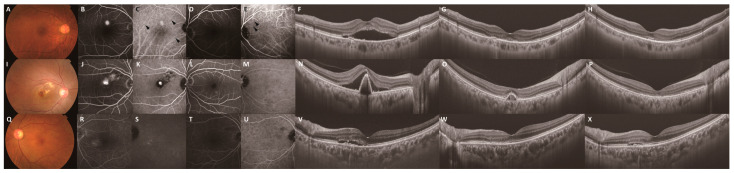
Representative multimodal imaging findings during the treatment course in patients with nAMD. Images (**A**–**H**) show multimodal images of a 77-year-old woman in the IVF and PH groups. Images (**I**–**P**) show multimodal images of a 54-year-old woman in the IVF and non-PH group. Images (**Q**–**X**) show multimodal images of a 73-year-old woman in the IVA and non-PH group. Images (**A**–**F**,**I**–**N**,**Q**–**V**) were obtained at the start of treatment, and images (**G**,**O**,**W**,**H**,**P**,**X**) were obtained 2 months, 1 year, and 6 months after the third loading injections, respectively. (**A**) Color fundus photograph showing scattered yellowish drusenoid deposits over the temporal upper vascular arcade. (**B**) Late-phase FA image showing an area of hyperfluorescence in the upper fovea. (**C**) Late-phase ICGA image showing hyperfluorescence of the abnormal choroidal vessels corresponding to the leakage area in the FA image, and scattered PH (arrowheads) over the temporal upper vascular arcades. (**D**) Late-phase FA image of the fellow eye showing no obvious abnormalities. (**E**) Late-phase ICGA image showing the PH (arrowhead) around the fovea. (**F**) B-scan with a swept-source OCT image of the macula showing a flat irregular PED with SRF. (**G**,**H**) B-scan with a swept-source OCT image of the macula showing no SRF. (**I**) Color fundus photograph showing a hemorrhage surrounded by hard exudates in the fovea. (**J**) Late-phase FA image showing the area of hyperfluorescence corresponding to the hemorrhage. (**K**) Late-phase ICGA image showing hyperfluorescence of abnormal choroidal vessels with polypoidal lesions corresponding to the leakage area in the FA image. (**L**,**M**) Late-phase FA and ICGA images of the fellow eye showing no obvious abnormalities. (**N**) B-scan with a swept-source OCT image of the macula showing a steep PED with SRF. (**O**,**P**) B-scan with a swept-source OCT image of the macula showing no SRF. (**Q**) Color fundus photograph showing a ring-shaped yellowish-white lesion nasal to the fovea. (**R**) Late-phase FA image showing an area of hyperfluorescence corresponding to the yellowish-white lesion. (**S**) Late-phase ICGA image showing hyperfluorescence of abnormal choroidal vessels corresponding to the leakage area in the FA image. (**T**,**U**) Late-phase FA and ICGA images of the fellow eye showing no obvious abnormalities. (**V**) B-scan with a swept-source OCT image of the macula showing a flat irregular PED with SRF. (**W**) B-scan with a swept-source OCT image of the macula showing no SRF. (**X**) B-scan with a swept-source OCT image of the macula showing a flat irregular PED with SRF. FA, fluorescein angiography; ICGA, indocyanine green angiography; MNV, macular neovascularization; OCT, optical coherence tomography; PED, pigment epithelial detachment; PH, punctate hyperfluorescence; SHRM, subretinal hyperreflective material; SRF, subretinal fluid.

As prespecified, the primary endpoint was the 1-year retreatment rate after completion of the loading dose regimen within PH and non-PH groups. In the PH group, the retreatment rate within 1 year after completion of the loading dose regimen was 57.1% (16/28 eyes) in the IVF group and 64.3% (18/28 eyes) in the IVA group. The difference between the two groups was not statistically significant (log-rank *p* = 0.60; HR = 0.84, 95% CI: 0.43–1.66, *p* = 0.63; Figure 4A). In the non-PH group, the retreatment rate within 1 year after completion of the loading dose regimen was 71.4% (20/28 eyes) in the IVF group and 92.9% (26/28 eyes) in the IVA group. The IVF group had a lower 1-year retreatment rate than the IVA group (log-rank *p* = 0.004; HR = 0.49, 95% CI: 0.27–0.88, *p* = 0.017; Figure 4B). Representative cases are shown in Figure 5.

## 4. Discussion

This study compared the treatment outcomes of IVF and IVA in treatment-naïve patients with nAMD, with baseline characteristics adjusted using propensity score matching. In the PH group, no significant differences in the treatment efficacy were observed between the two agents. In contrast, in the non-PH group, IVF was associated with a significantly higher no retinal fluid rate and a lower retreatment rate after the loading dose regimen compared with IVA. To the best of our knowledge, no previous study has identified a specific subgroup of patients with nAMD that derived superior therapeutic benefits from IVF. These findings suggest that the underlying pathophysiology of nAMD differs according to the presence or absence of PH and that Ang-2 may play a more prominent role in disease mechanisms in patients with nAMD without PH.

Several studies have compared treatment outcomes between IVF and IVA. A post hoc analysis of the TENAYA and LUCERNE trials demonstrated that 1 month after the third loading injection, visual outcomes were comparable between the two agents; however, the frequency of fluid resolution was higher in the IVF group than in the IVA group [17]. Similarly, Fukuda et al. reported comparable visual outcomes but a higher fluid resolution rate in the IVF group than in the IVA group 1 month after the initial injection [18]. The present findings are consistent with previous observations. In contrast, Hara et al. reported that the rate of fluid resolution 2 months after the initial injection was higher in the IVA group than in the IVF group [19]. A potential explanation for this discrepancy is that the TENAYA and LUCERNE trials were phase III randomized controlled trials and the study by Fukuda et al. used propensity score matching to adjust for baseline characteristics, whereas the study by Hara et al., although showing no statistically significant baseline differences, did not adjust for patient background using such methods.

In both the IVF and IVA groups in this study, anatomical improvements from baseline to month 4 were observed; however, no significant gain in BCVA was detected. This differs from Fukuda et al., who reported concomitant improvements in anatomy and vision at month 3 in both groups [18], and from Hara et al., who observed anatomical improvements in both groups but a significant BCVA gain only in the IVA group by month 3 [19]. The discrepancy in our cohort may reflect irreversible photoreceptor damage caused by pre-treatment intraretinal/subretinal fluid or hemorrhage, which can blunt short-term visual recovery despite morphological normalization. It may also be region- or case-mix–related, including differences in baseline disease phenotype and management patterns.

The pathophysiology of nAMD comprises two major subtypes: drusen-associated nAMD and pachychoroid-associated nAMD [20]. Drusen are extracellular deposits that accumulate between the RPE and Bruch’s membrane and are composed of various inflammatory components, including cholesterol [21], apolipoprotein E (ApoE) [22], and malondialdehyde [23]. Previous studies have shown that the concentration of inflammatory cytokines in the aqueous humor of patients with PNV is lower than that in patients with nAMD [24], suggesting that chronic inflammation plays a central role in the pathogenesis of drusen-associated nAMD. Although the precise pathogenesis of pachychoroid-associated nAMD remains unclear, it has been hypothesized that venous congestion and impaired choroidal outflow lead to the development of dilated choroidal vessels (pachyvessels) that subsequently compress the choriocapillaris. This compression is believed to cause ischemic damage to the RPE, thereby playing a central role in disease progression [25]. PH is strongly associated with CVH, a characteristic feature of pachychoroid spectrum diseases, and is frequently observed in CSC and PCV [11,12]. Based on these observations, the PH group in the present study was considered to represent pachychoroid-associated nAMD, whereas the non-PH group likely corresponded to drusen-associated nAMD.

Although elevated levels of Ang-2 in the aqueous humor of patients with nAMD have been reported [26], no significant difference in treatment efficacy between IVF and IVA was observed in the PH group. PNV is characterized by MNV accompanied by pachyvessels in the Haller’s layer, attenuation of the choriocapillaris and choroidal vessels in the Sattler’s layer. These findings suggest that the underlying pathology in the PH group may be primarily VEGF-driven, mediated by ischemic mechanisms via the hypoxia-inducible factor-1α (HIF-1α) signaling pathway. An in vitro study using bovine retinal endothelial cells demonstrated that VEGF stimulation directly increases Ang-2 mRNA expression [27]. Furthermore, Ang-2 promoted angiogenesis in the presence of VEGF and inhibited angiogenesis in the absence of VEGF [28]. Therefore, in the PH group, in which VEGF upregulation due to ischemia likely played a central role, VEGF inhibition may have secondarily suppressed the angiogenic activity of Ang-2. This mechanism may account for the comparable treatment outcomes of IVF and IVA observed in this study.

In contrast, the non-PH group demonstrated superior treatment outcomes with IVF compared with IVA, suggesting a disease mechanism driven primarily by the inflammation-induced expression of VEGF and Ang-2. Drusen contain various inflammatory components such as ApoE [22] and malondialdehyde [23], which have been reported to activate the complement pathway and promote the production of inflammatory cytokines including interleukin-6 (IL-6). Multiple studies have demonstrated that IL-6 induces VEGF expression via the JAK/STAT3 signaling pathway, thereby contributing to angiogenesis and tumor invasion [29]. In the ophthalmic field, IL-6 has also been shown to promote the development of MNV in a laser-induced MNV mouse model [30]. Moreover, IL-6 reduced angiopoietin-1 (Ang-1) levels. In an in vitro co-culture model using rheumatoid arthritis synovial fibroblasts and human umbilical vein endothelial cells, IL-6 stimulation increased VEGF expression and decreased Ang-1 expression, whereas VEGF stimulation alone did not alter Ang-1 levels [31]. Ang-1 and Ang-2 bind to Tie2 receptors. Ang-1 activates Tie2 signaling and stabilizes blood vessels, whereas Ang-2 antagonizes this signaling pathway, contributing to vascular destabilization. The balance between Ang-1 and Ang-2 levels plays a critical role in the pathogenesis of vascular diseases. Therefore, we hypothesized that an imbalance in Ang-1/Ang-2 signaling, caused by increased VEGF and decreased Ang-1, plays a central role in the pathogenesis of drusen-associated nAMD. Under such conditions, direct inhibition of Ang-2 by IVF may have more effectively suppressed retinal fluid in the non-PH group, as demonstrated in the present study.

The present study had several limitations. First, the sample size was relatively small, and the study was retrospective. To address potential selection bias, we employed propensity score matching to balance the patient characteristics. Previous studies have suggested that the effectiveness of anti-VEGF therapy may vary with patient age. For example, Kurada et al. reported an association between fluid recurrence after the loading dose regimen and advanced age in patients with nAMD [32]. Furthermore, baseline visual acuity was identified as a predictor of long-term visual outcomes [33]. Therefore, propensity score matching was performed using age, baseline visual acuity, and PH status to align the IVF and IVA groups. However, unreported prognostic covariates relevant to anti-VEGF therapy were not included in this model; thus, residual confounding between matched groups cannot be excluded and effect estimates should be interpreted cautiously. In addition, with respect to the results in the PH group, baseline visual acuity was relatively better compared with typical nAMD cohorts, and a slight imbalance in the distribution of SHRM subtypes remained between two groups. These factors should be taken into account when interpreting the present findings. Our findings support the hypothesis that PH and non-PH subtypes differ in their pathophysiology, possibly due to an imbalance in Ang-1/Ang-2, but further large-scale studies are needed to confirm our results. Second, although we controlled local multiplicity within endpoints using Tukey–Kramer, we did not implement a global multiplicity adjustment across endpoints and subgroups; therefore, results beyond the prespecified primary endpoint should be interpreted as exploratory. Third, the levels of intraocular cytokines such as VEGF, Ang-2, and IL-6, were not measured directly. Future research should include prospective studies incorporating quantitative analyses of inflammatory and angiogenic factors in intraocular fluids to elucidate the underlying mechanisms of treatment response. Fourth, we assessed PH in the fellow eye because evaluation in the affected eye is often hindered by hemorrhage and other disease-related changes that can mask PH. Consistent with this rationale, Park et al. evaluated both eyes in unilateral nAMD and reported higher PH detection in the fellow eye (PCV 86.7%, AMD 60.0%) than in the affected eye (PCV 86.0%, AMD 40.0%) [12]. Nevertheless, we acknowledge the possibility of misclassification, and we interpret PH-based subgroup findings with appropriate caution.

## 5. Conclusions

In conclusion, no significant difference in treatment efficacy was observed between IVF and IVA in patients with nAMD and PH. In contrast, IVF demonstrated superior anatomical outcomes compared with IVA in patients without PH. This disparity in treatment response suggests that the underlying pathophysiology of nAMD with and without PH differs, particularly with respect to the mechanisms driving Ang-1/Ang-2 imbalance. However, these observations are hypothesis-generating and should not be applied to clinical stratification at this stage. Prospective, adequately powered, multicenter studies with standardized imaging and treatment protocols are required to clarify whether PH meaningfully modifies treatment response.

## Figures and Tables

**Table 1:** Clinical characteristics of patients treated with IVF and IVA.

Characteristic	PH Group			Non-PH Group		
	IVF	IVA	*p*	IVF	IVA	*p*
Number of eyes	28	28		28	28	
Age (years), mean (SD)	69.6 (10.0)	69.2 (6.8)	0.21	75.0 (8.4)	74.7 (8.3)	0.33
Sex (female), no. (%)	11 (39.3)	7 (25.0)	0.09	11 (39.3)	9 (32.1)	0.58
Hypertension, no. (%)	15 (53.6)	17 (60.7)	0.59	17 (60.7)	13 (46.4)	0.28
Diabetes, no. (%)	2 (7.1)	4 (14.3)	0.38	7 (25.0)	9 (32.1)	0.55
Smoking history (ever-smoker), no. (%)	17 (60.7)	21 (75.0)	0.59	18 (64.3)	18 (64.3)	1.00
Presence of SHRM, no. (%)			0.06			0.92
Exudation	4 (14.3)	0 (0.0)		4 (14.3)	0 (0.0)	
Hemorrhage	2 (7.1)	4 (14.3)		2 (7.1)	4 (14.3)	
Neovascular tissue	0 (0.0)	0 (0.0)		0 (0.0)	0 (0.0)	
No SHRM	22 (78.6)	24 (85.7)		22 (78.6)	24 (85.7)	
Presence of IRF, no. (%)	4 (14.3)	4 (14.3)	1.00	6 (21.4)	4 (14.3)	0.48
Presence of SRF, no. (%)	26 (92.9)	28 (100)	0.09	26 (92.9)	28 (100)	0.09
Presence of polypoidal lesion, no. (%)	14 (50.0)	10 (35.7)	0.28	5 (17.9)	7 (25.0)	0.51

PH, punctate hyperfluorescence; IVF, intravitreal faricimab; IVA, intravitreal aflibercept; SHRM, subretinal hyperreflective material; IRF, intraretinal fluid; SRF, subretinal fluid.

**Table 2:** The time course of BCVA, CRT, and SFCT in the loading dose regimen.

Group	Outcome	IVF	IVA	Drug, *p*	Month, *p*	Drug × Month, *p*
PH group	logMAR			0.55	0.003	0.75
	baseline, LS mean (95% CI)	−0.02 (−0.07–0.04)	0.00 (−0.06–0.05)			
	1M, LS mean (95% CI)	−0.02 (−0.07–0.04)	−0.01 (−0.06–0.04)			
	2M, LS mean (95% CI)	−0.07 (−0.11–0.02)	−0.04 (−0.09–0.01)			
	4M, LS mean (95% CI)	−0.07 (−0.11–0.03)	−0.04 (−0.09–0.00)			
	CRT (μm)			0.13	<0.001	0.09
	baseline, LS mean (95% CI)	292.6 (264.8–320.5)	325.5 (297.7–353.3)			
	1M, LS mean (95% CI)	247.8 (223.9–271.7)	256.7 (232.8–280.6)			
	2M, LS mean (95% CI)	223.5 (199.3–247.7)	246.2 (222.0–270.4)			
	4M, LS mean (95% CI)	232.5 (207.2–257.7)	246.9 (221.6–272.1)			
	SFCT (μm)			0.47	<0.001	0.06
	baseline, LS mean (95% CI)	284.2 (239.1–329.3)	261.3 (216.2–306.4)			
	1M, LS mean (95% CI)	274.0 (229.7–318.3)	250.9 (206.6–295.1)			
	2M, LS mean (95% CI)	263.9 (218.2–309.7)	244.4 (198.7–290.2)			
	4M, LS mean (95% CI)	269.5 (222.1–316.9)	242.0 (194.6–289.4)			
Non-PH group	logMAR			0.81	0.51	<0.001
	baseline, LS mean (95% CI)	0.38 (0.27–0.49)	0.38 (0.26–0.49)			
	1M, LS mean (95% CI)	0.36 (0.19–0.52)	0.41 (0.25–0.57)			
	2M, LS mean (95% CI)	0.37 (0.22–0.52)	0.36 (0.21–0.51)			
	4M, LS mean (95% CI)	0.42 (0.28–0.57)	0.30 (0.15–0.45)			
	CRT (μm)			0.69	<0.001	0.04
	baseline, LS mean (95% CI)	302.9 (280.2–325.5)	282.9 (260.3–305.5)			
	1M, LS mean (95% CI)	210.7 (192.0–229.4)	219.6 (200.9–238.4)			
	2M, LS mean (95% CI)	207.7 (188.0–227.4)	227.2 (207.5–246.9)			
	4M, LS mean (95% CI)	218.6 (198.1–239.2)	230.2 (209.7–250.7)			
	SFCT (μm)			0.15	<0.001	0.03
	baseline, LS mean (95% CI)	172.4 (136.6–208.1)	224.7 (188.9–260.5)			
	1M, LS mean (95% CI)	167.4 (128.7–206.2)	203.4 (164.6–242.1)			
	2M, LS mean (95% CI)	163.6 (125.2–202.0)	194.6 (156.2–233.0)			
	4M, LS mean (95% CI)	155.8 (117.9–193.6)	192.1 (154.2–229.9)			

PH, punctate hyperfluorescence; IVF, intravitreal faricimab; IVA, intravitreal aflibercept; M, month; LS mean, least squares mean; CI, confidence interval; *p* values are Type III tests of fixed effects (REML, Satterthwaite df).

## Data Availability

The data used to support the findings of this study are restricted by the Kawasaki Medical School Ethics Committee to protect patient privacy. Data are available from Hiroyuki Kamao [hironeri@med.kawasaki-m.ac.jp] for researchers who meet the criteria for access to confidential data.
